# Late diagnosis of congenital diaphragmatic hernia: a case report

**DOI:** 10.1186/s13256-023-03987-x

**Published:** 2023-06-12

**Authors:** Naomi A. Mwamanenge, Fatima Mussa, Masawa K. Nyamuryekung’e, Martha Mkony, Yaser Abdallah, Karim Manji

**Affiliations:** 1 Muhimbili University of Health and Allied Sciences (MUHAS), P. O box 65001, Dar es Salaam, Tanzania; 2 Muhimbili National Hospital (MNH), Dar es Salaam, Tanzania; 3 Aga Khan Hospital, Dar es Salaam, Tanzania

**Keywords:** Congenital diaphragmatic hernia, Pneumonia

## Abstract

**Background:**

Congenital diaphragmatic hernia beyond the neonatal period is not uncommon. Its diagnosis in infancy and early childhood poses a challenge owing to different clinical presentation ranging from gastrointestinal to respiratory symptoms. These neonates are usually misdiagnosed as having pneumonia until radiological imaging picks up the defect during routine scan for worsening respiratory symptoms. In high-income countries, the survival rate for these patients has been reported to be high, while in Sub-Saharan Africa the survival rate is still low due to delayed diagnosis, delayed referral, and hence delayed management.

**Case report:**

We present an African male baby from non-consanguineous parents, 6 weeks old, diagnosed with congenital diaphragmatic hernia at 6 weeks of age after failure to respond to antibiotics for suspected pneumonia. Despite attempts at management, he died at 5 weeks post surgery.

**Conclusion:**

Our case emphasizes the importance of early clinical suspicion and early detection for a differential diagnosis of congenital diaphragmatic hernia in infants who present with respiratory symptoms not responding to antibiotics or recurrent pneumonia, and improving the availability of imaging in primary care facilities to diagnose such defects early and manage them accordingly.

## Introduction

Congenital diaphragmatic hernia (CDH) is a well-recognized entity occurring in 1 out of 25,000 to 30,000 babies [1]. These hernias can be detected antenatal or after delivery. Management can start in utero by selecting cases suitable for fetal therapy. In centers or countries where fetal therapy is not possible, mothers are referred to tertiary care center for pre- and post-natal management. The timing of delivery is crucial as it predicts outcome, and those delivered beyond 38 weeks had favorable outcome [2]. Babies usually present with symptoms of respiratory distress shortly after birth, although symptoms may delay to a few days depending on the extent of herniation of abdominal content into the thorax. Surgery is delayed for 48–72 h, allowing stability from pulmonary hypertension. The majority of affected infants are male babies [2]. Late-onset CDH beyond the neonatal period poses a significant diagnostic challenge. Symptoms comprise a wide range from mild to moderate gastroesophageal reflux to respiratory distress not responding to usual medication/care or incidentally during routine X-ray examination for suspected pneumonia [3]. In Sub-Saharan Africa, the exact incidence of the disease is not known, however, owing to late presentation and lack of imaging in most countries, and misdiagnosis makes it a major challenge for management, leading to poor outcomes. In our settings, there is still a challenge owing to lack of imaging in most places, making clinicians rely on physical findings and examination to diagnose most cases. However, a good physical examination with additional imaging can decrease time to diagnosis and thereafter initiate proper management and decrease mortality.

## Case report

We present a case of congenital diaphragmatic hernia diagnosed at 6 weeks of life, referred to us from up-country. This male baby was referred to our center with respiratory distress and chest X-ray (CXR) with suspected diaphragmatic hernia.

This is an African male baby delivered term via spontaneous vaginal delivery (SVD) from non-consanguineous parents, with a birth weight of 3.6 kg and Apgar score of 7 and 9 in 1st and 5th minute. He was well, received the initial vaccines (BCG and OPV) and was discharged home 24 h after delivery.

His mother was aged in her early 30s, para 5 + 0; during this pregnancy she started her antenatal clinic visits at 7 months of age due to lower abdominal pain. She had negative serology for HIV and VDRL, and received hematinic and multivitamins. Her antenatal ultrasound was unremarkable, however anomaly scans are rarely done in our country as most ultrasonographers do not have the expertise to do them.

**Fig. 1:**
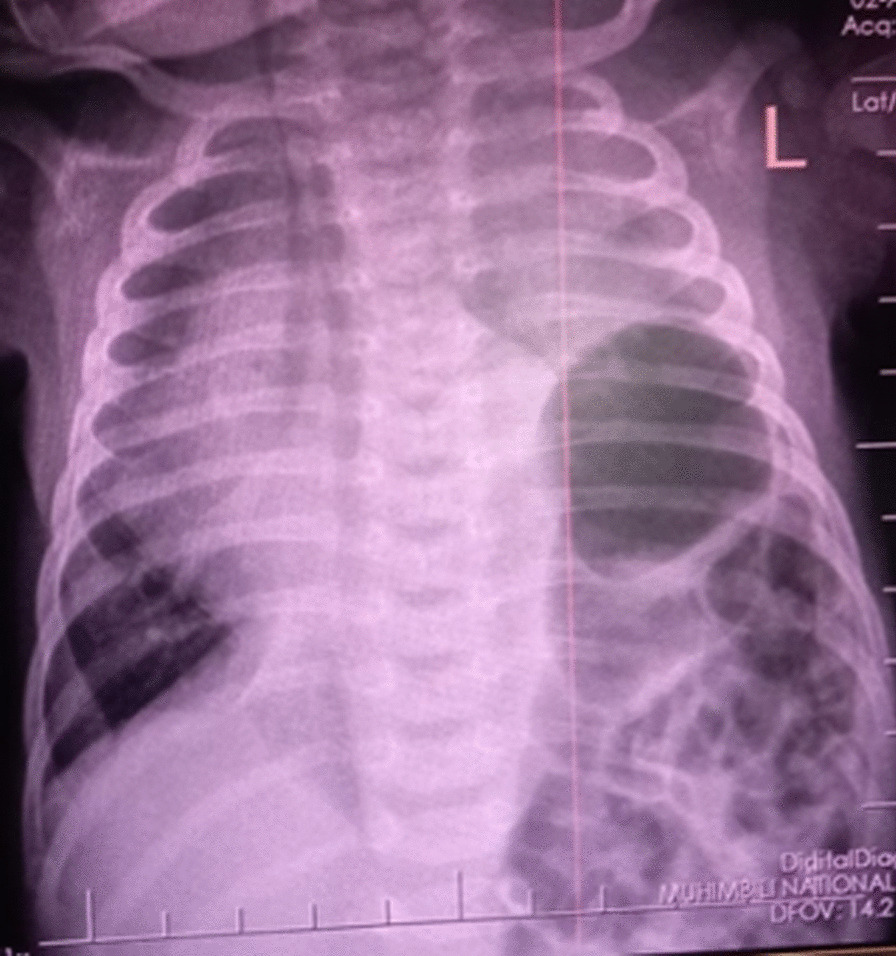
Chest X-ray showing herniated bowel loops into the left hemithorax with mediastinal right shift

At the 5th week of life, the baby started having difficulties in breathing, with audible wheeze. He was taken to a nearby hospital, where initially therapy with oxygen was instituted and treatment for suspected pneumonia was initiated. However, his condition worsened, and he was then transferred to level 2 hospitals for further diagnosis and treatment due to lack of imaging. Upon arrival, chest X ray was done, which revealed herniation of bowel loops into the left hemithorax with right mediastinal shift with normal right hemithorax (Fig. 1), giving a diagnosis of congenital diaphragmatic hernia. Baby was referred to tertiary hospital for surgical management.

On admission, the baby weighed 3 kg and had moderate respiratory distress with peripheral cyanosis. The respiratory rate was 88 breath/min, oxygen saturation SpO_2_ of 85%, and heart rate 167 beats/min, with blood pressure of 82/52 (60). There was decreased breath sounds on the left side with a slight scaphoid abdomen. His neurological examination was normal.

During surgery, a left posterolateral diaphragmatic defect was noted, made of sac of peritoneum parietal layers with small lung sequestration; contents were spleen, small bowel, and left lobe of liver.

**Fig. 2:**
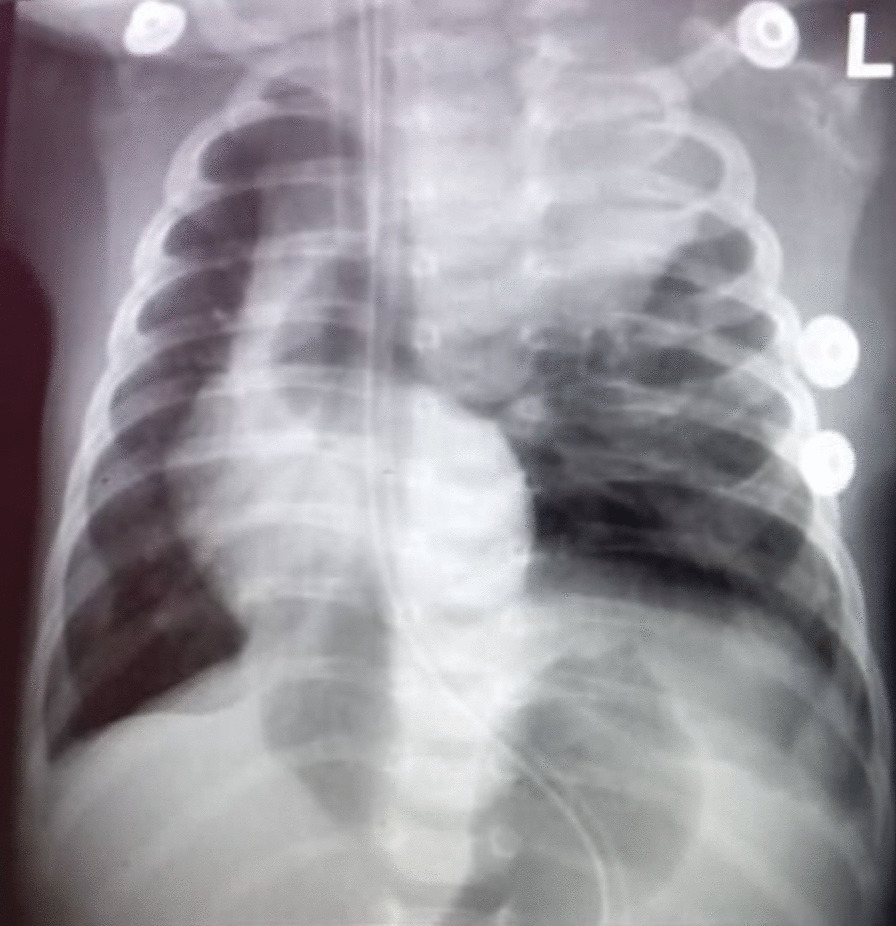
Chest X-ray done a few hours post-surgery, showing right mediastinal shift and left upper lobe of the lung with hypoplasia

**Fig. 3:**
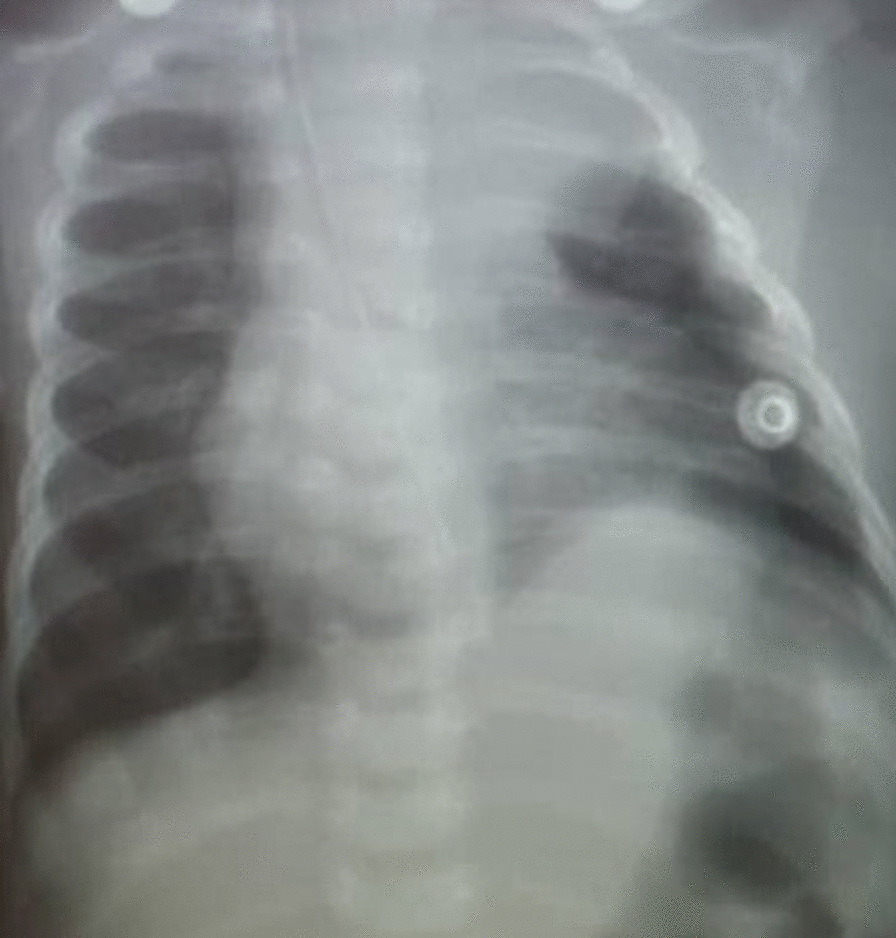
Chest X-ray done 48 h post-surgery, showing left upper lobe lung hypoplasia

After surgery, the baby was kept on a convectional mechanical ventilator with lung protective mechanics. He has been progressing well, and feeding was initiated on the third day post surgery. Chest X-rays done immediately after surgery (Fig. 2) and 48 h later showed mediastinal shift to the right with hypoplasia of the left upper lobe (Fig. 3).

The baby stayed in the neonatal intensive care unit for a period of 5 weeks, where he received total parental nutrition and gradually started on oral milk feeds. He was also given post-surgical intravenous antibiotics, ceftriaxone for 7 days. His surgical wound had gaped, and revision was done on the 10th day post-surgery. At the 3rd week, he developed excessive oral secretion and multiple endotracheal tube blockages necessitating multiple re-intubations. We were unable to wean off from mechanical ventilator due to worsening work of breathing. He then had fever spikes. A CRP protein of 140 mg/dl and blood cultures were taken using aseptic techniques; 2 ml was drawn and kept in aerobic culture bottles, and the sample was taken immediately to the central pathology laboratory using a special transport container. Gram-negative bacteria were isolated, *Klebsiella pneumonia* sensitive to meropenem. Gram-negative sepsis was diagnosed. Intravenous meropenem was started, given 8 hourly. However, at the 5th week post-surgery, his condition worsened and the baby died.

## Discussion

Congenital diaphragmatic hernia is a condition in which there is herniation of abdominal viscera into the thoracic cavity. Incidence is around 1 in every 30,000 live births, with a predilection for males [2]. Owing to the thoracic cavity being occupied by abdominal viscera, there is high association of pulmonary hypoplasia and pulmonary hypertension. The degree of lung hypoplasia and pulmonary hypertension are key determinants of the morbidity and mortality of these patients [4].

Whereas the majority of cases of CDH are identified in the neonatal period, late diagnosis has been described. Late diagnosis of congenital diaphragmatic hernia is defined as diagnosis beyond the neonatal period or as an incidentally finding of herniated bowel loops into the thoracic cavity on routine chest X-ray examination. The incidence for late diagnosis is variable, ranging from 3% to 5% [5]. Infants with late presentation can have variable symptoms, ranging from respiratory to gastrointestinal symptoms [6, 7].

The patient presented herein is a 7-week-old baby who first presented with increased work of breathing at 5 weeks of age; treatment with low-flow oxygen therapy and antibiotics for presumed pneumonia prior to referral to higher center did not help the baby. Challenges in imaging including the availability of trained ultrasonographers for anomaly scans can lead to delays in diagnosing these babies, and a delay to plan where delivery should take place for proper management. In our setting, a lack of chest X-ray at most centers may cause high mortality and morbidity due to significant delays in diagnosis.

Congenital diaphragmatic hernia can be diagnosed antenatally [8]. Once antenatal diagnosis is made, proper planning for delivery including referral to a tertiary center with facilities for cardiopulmonary support and stabilization of the baby (at birth, elective intubation and gastric tube insertion is practiced to prevent air in the bowel compromising lung expansion) employed as surgery is awaited [9]. Unfortunately, our patient started antenatal clinic at 7 months of age, and antenatal ultrasound was unremarkable.

The baby was well at birth but developed acute respiratory distress at 5 weeks of age, which worsened with time. The care for these infants has evolved from previous aggressive surgical approach to clinical stabilization of the patient as we wait for pulmonary hypertension to resolve at least within 72 h or longer if needed [10, 11]. The surgical techniques are variable and can range from thoracoscopic repair to open repair via subcostal incision, whereby herniated abdominal organs are returned to the abdominal cavity with consequently diaphragmatic closure [8, 11, 12]. Our patient underwent a left subcostal incision, whereby the defect was noted with the contents that were herniated, reduction was done with subsequent tension-free closure.

The foramen where the hernia occurs classifies CDH, whereby the commonest types are Bochdalek and Morgagni. They can be unilateral or bilateral. The most common is the Bochdalek type. The Bochdalek type has a posterolateral position, which can result in mediastinal shift on the contralateral side and pulmonary hypoplasia of the same side of the hernia [13, 14]. Our patient had Bochdalek hernia with herniation of bowel loops, spleen, and part of the liver into the thoracic cavity. Post-surgery chest X-ray revealed lung hypoplasia in upper left lobe of the lung.

The degree of pulmonary hypoplasia and pulmonary hypertension predicts the prognosis of these infants [15]. It is favorable if diagnosed early, especially during antenatal screening, hence plans for delivery are sought with lung-protective mechanics in place [16, 17]. Several follow-up studies on survivors have been done. Some report a degree of recurrent respiratory infections and an evidence of obstructive and/or restrictive airway disease. Others report recurrence of the hernia on the same side [18]. There can be neurodevelopmental impairment with both somatic and social problems when compared with controls. Gastroesophageal reflux has also been reported, with resulting failure to thrive. Musculoskeletal changes can also occur including pectus deformity [19].

## Conclusion

With the evolution of antenatal diagnosis and postnatal care of infants with CHD in high-income countries, prognosis is favorable, being up to 80% from the previous 50%. Even in case of delayed diagnosis of these neonates beyond the neonatal period, prognosis is still favorable. In Sub-Saharan Africa, diagnostic challenges still prevail due to a lack of imaging facilities in primary centers and the expertise to pick up the defect in utero. Timely antenatal visits and proper antenatal scans from an expert can lead to detection of these defects early and therefore a plan for delivery and care can be arranged early.

## Data Availability

Not applicable.
